# Analysis of Surgical Procedures on the Forearm and Hand and Their Relationship with Complex Regional Pain Syndrome: A Cross-sectional Study

**DOI:** 10.1055/s-0044-1785659

**Published:** 2024-06-22

**Authors:** Caio Carvalho dos Santos Souza, João Miguel Casado Neto, Manoel Vítor Maciel Bocchi, Danielle Horing Grubert

**Affiliations:** 1 Departamento de Ortopedia e Medicina da Dor, Hospital Bom Samaritano, Maringá, PR, Brasil; 2 Departamento de Ortopedia e Cirurgia da Mão, Hospital Bom Samaritano, Maringá, PR, Brasil; 3 Departamento de Ortopedia e Traumatologia, Associação Beneficente Bom Samaritano, Maringá, PR, Brasil

**Keywords:** carpal tunnel syndrome, complex regional pain syndromes, hand injuries, nerve compression syndromes, orthopedic precedures

## Abstract

**Objective** Complex regional pain syndrome (CRPS) requires further understanding. Thus, the present study aimed to analyze if pre- and intraoperative factors may be related to the development of CRPS in the postoperative period.

**Methods** We reviewed 1,183 medical records of patients undergoing forearm and hand surgeries from 2015 to 2021. The data of interest, that is, diagnosis, incisions, synthesis material, and anesthesia, were collected, tabulated, and statistically analyzed, with subsequent calculation of the odds ratios.

**Results** Most patients were female, aged between 30 and 59 years, and sought the service electively (67% of the cases). The diagnoses included soft tissue trauma (43%), bone trauma (31.6%), and compressive syndromes (25.5%). During this period, 45 (3.8%) subjects developed CRPS. The statistical analysis showed that the chance of developing CRPS is twice as high in patients with compressive syndrome, especially carpal tunnel syndrome (CTS), which represented most surgeries performed in our service (24%). Two or more incisions occurred in 7.6% of the cases, which tripled the chance of developing postoperative CRPS. Gender, age, use pf synthetic material, type of anesthesia type did not statistically increase the risk of developing postoperative CRPS.

**Conclusion** In short, the incidence of CRPS is low; however, it is critical to know and recognize the risk factors for prevention and active screening in the postoperative period.

## Introduction

Complex regional pain syndrome (CRPS) is a clinical condition characterized by persistent pain disproportionate to the injury, with changes in sensitivity, motricity, and vascularization. If the condition persists, it leads to skin changes and atrophy in the affected area. 1 2 3 It occurs predominantly in the peripheral region of the upper limbs, 2 but it can affect any portion of the body. 1 2 3 4 Its etiology is not entirely understood. The diagnosis is clinical, based on the Budapest criteria ( Table 1 ), and, to date, there are no specific supplementary tests. 1 2 3 The syndrome often occurs after trauma, surgery, or conditions affecting the local nervous system, 2 3 4 and it is 2 to 4 times more common in women aged between 20 and 50 years. 1 2 3 4 Patients undergoing surgery are more susceptible to developing CRPS than those not undergoing surgery. 3 5 Therefore, the objective of the present study was to analyze whether interventions performed during the surgical procedure to treat the main conditions in the routine of the hand surgery outpatient clinic contribute or not to the development of postoperative CRPS.

**Table 1:** Budapest clinical criteria for complex regional pain syndrome

**Continuous pain disproportional to any trigging event in addition to at least one symptom reported by the patient in three of the following four categories, and symptoms from at least two categories evidenced by the doctor during the visit**
** Sensory: Reports of hyperalgesia, allodynia, or both**
** Vasomotor: Reports of temperature difference, change in skin color, or both**
** Sudomotor/edema: Reports of edema, changes in sweating, and/or sweating asymmetry**
** Motor/trophic: Reports of decreased range of motion, motor dysfunction (weakness, tremor, dystonia), and/or trophic changes (hair, nail, skin)**
**Lack of other diagnoses that better explain the signs and symptoms**

## Materials and Methods

The Ethics Committees of the two institutions where the data were collected approved the present study, which complied with institutional guidelines and Resolution no. 466/20112 of the Brazilian National Health Council. The study was cross-sectional, observational, retrospective, and included all patients who underwent a surgical procedure from January 2015 to December 2021. These surgeries were performed by a hand surgeon who is a member of an orthopedics team working at a regional healthcare unit. After hospital discharge, patients were referred to the outpatient clinic for follow-up with the same surgeon to ensure consistency in the clinical analysis. This process is especially relevant in CRPS due to its diverse signs and symptoms and the current lack of a gold-standard diagnosis, leading to the need for uniform clinical assessments throughout medical follow-up. The diagnosis was based on the Budapest criteria, including constant pain inconsistent with any triggering event accompanied by other signs and symptoms, as outlined in Table 1 .

Among all operated patients, we selected those diagnosed with CRPS at least 30 days after surgery. Patients with conditions treated non-surgically, duplicate records in the system, or subjects with complications but who had not undergone surgery during the preestablished period were excluded from the study.

Data were collected exclusively by analyzing medical records. Details such as gender, age, year of admission, nature of the admission (elective or emergency), and general and specific diagnoses were recorded, as well as specific information about the surgical procedure, including number of incisions, synthesis materials, type of anesthesia administered during the procedure, and the subsequent development (or not) of postoperative CRPS.

Data tabulation was made through the Excel (Microsoft Corp., Redmond, WA, United States), version 2013, software. Next, we performed the statistical analysis, that is, application of the Chi-squared test followed by univariate logistic regression analysis. We included variables with statistically significant results in the multivariate analysis to identify whether there was an independent association with CRPS. The results were expressed as odds ratios (ORs) and 95% confidence intervals (95%CIs), and OR was established when the *p* -value was lower than 0.05 ( *p* < 0.05). All these statistical analyses used the R application (R Foundation for Statistical Computing, Vienna, Austria), version 3.8.

Quantitative data were expressed as frequencies and percentages to facilitate understanding. Regarding qualitative information, the most significant values are mentioned in the text, and general data are shown in a table for better analysis and visualization during the discussion.

## Results

During the analyzed period, 1,183 subjects underwent surgery, including 770 (65.1%) women and 413 (34.9%) men. The overall mean age was of 49.6 ± 17 years. According to the Budapest criteria, 45 (3.8%) patients, with a mean age of 53.02 ± 13 years, developed CRPS during the postoperative follow-up.

Regarding the nature of the admission, most patients sought the service electively, resulting in 795 procedures (67.2%), while emergency surgeries, mainly resulting from fractures, totaled 388 cases (32.8%).

Patients undergoing surgical procedures were categorized according to the nature of the condition: soft tissue injuries (507; 42.9%), fractures (374; 31.6%), and compressive syndromes (302; 25.5%). This last set included carpal tunnel syndrome (CTS), accounting for 284 (24%) procedures, and ulnar nerve compression, with 18 (1.5%) interventions. The most frequently performed procedures after CTS were fractures of the forearm bones, mainly the distal radius, with 267 surgeries (22.6%), and fractures of the hand bones, with 110 (9.3%) cases.

Regarding the number of incisions performed on the same patient for treatment, most subjects (1,093; 92.4%) required a single surgical approach. On the other hand, 90 (7.6%) cases required 2 or more incisions.

As for the fixation material, 753 (63.7%) cases did not use specific rigid synthetic material, mostly including decompressions and tenorrhaphies. The materials most frequently used in the surgeries were Kirschner wires in 240 (20.3%) cases, and volar plates in 126 (10.7%) cases.

Regarding the choice of anesthesia, intravenous general anesthesia predominated, totaling 607 (51.3%) procedures, closely followed by plexus block in 576 (48.7 %) patients.

The statistical analysis identified an increased risk of developing CRPS in the postoperative period. The presence of disease or compressive syndrome ( *p* = 0.0361), the specific diagnosis of CTS ( *p* = 0.0426), and the performance of 2 or more surgical incisions ( *p* = 0.0194) increased this risk. The multivariate logistic analysis showed that compressive syndrome (OR: 2; 95%CI: 1.09–3.69), CTS (OR: 1.98; 95%CI: 1.07–3.67), and the performance of 2 or more incisions (OR: 2.7; 95%CI: 1.26-6.18) alone increased the chance of developing postoperative CRPS. Table 2 summarizes the results.

**Table 2:** Surgical procedures performed and incidence of complex regional pain syndrome (CRPS)

		Patrients: n(%)	CRPS patients: n(%)	*p* -value (Chi-squared)	Odds ratio	95% confidence interval
**Condition that led to the surgical procedure**	Soft tissue lesions	507(42.9)	15(3)	0.074	−	−
	Fractures	374(31.6)	12(3.2)			
	Compressive syndromes	302(25.5)	18(6)	0.036	2	1.09–3.69
**Reason for the surgeries**	Carpal tunnel syndrome	284(24)	17(6)	0.042	1.98	1.07–3.67
	Forearm bone fracture	267(22.6)	11(4.1)	0.204	−	−
	Hand bone fracture	110(9.3)	1(0.9)			
	Trigger finger	98(8.3)	4(4.1)			
	Material removal/debridement	80(6.8)	0			
**Number of incisions**	1	1093(92.4)	37(3.4)	0.019	2.7	1.26–6.18
	≥ 2	90(7.6)	8(8.9)			
**Synthesis material**	None	753(63.7)	32(4.2)	0.722	−	−
	Kirschner wire	240(20.3)	6(2.5)			
	Volar plate	126(10.7)	6(4.8)			
	Dynamic compression plate	20(1.7)	1(5)			
	Herbert screw	20(1.7)	0			
**Anesthesia**	General intravenous anesthesia	607(51.3)	22(3.8)	1	1.01	0.56–1.83
	Plexus block	576(48.7)	23(3.8)			

## Discussion

Complex regional pain syndrome is a debilitating and complex condition mainly affecting the periphery of the upper limbs. It is predominantly unilateral and associated with sensory, vasomotor, and dystrophic changes. 1 2 4 It can be preceded by a local known lesion, but it is idiopathic in 10% of the cases. 1 3 5 6 The symptoms often begin 15 to 30 days after the injury, 2 7 the diagnosis is clinical, and, currently, there are no validated tests to confirm it. The literature reports divergent data regarding its incidence, with comprehensive estimates, 1 2 5 7 and regarding the risk factors, including comorbidities such as depression, fibromyalgia, rheumatoid arthritis, and metabolic disorders. 1 2 3 4 5

The development of CRPS occurs in two main phases, each with its features: in the acute or hot phase, proinflammatory mediators are released, resulting in symptoms such as paresthesia, hyperalgesia, anodynia, signs of vasomotor dysfunction, marked edema, and dyskinesia; 1 3 6 and the chronic or cold phase is characterized by activation of keratinocytes, fibroblasts, and osteocytes, leading to increased expression of adrenergic receptors and hyperstimulation of the sympathetic nervous system, which causes local vasoconstriction, with pale, cold, and sticky limbs. 1 3 6 The symptoms are often subjective and, if the process is not interrupted, it leads to alopecia, nail changes, muscle atrophy, and chronic pain. 1 2 6

Among the 1,138 subjects operated on during the 5 years of research, 45 (3.8%) presented postoperative CRPS. Both in the total group of operated subjects and among those who developed CRPS, most patients were women, with an average age of 49 to 53 years. 1 2 3 4 Although Harden et al. 1 and Dutton and Rhee, 3 as well as other authors, have highlighted the prevalence of CRPS in women, which is three times higher than in men, this gender association has not been shown to increase the risk of this complication. 1 3 7

Studies with multiple patients with CRPS, as mentioned by Taylor et al., 6 have identified the origin of this chronic pain, and they mention trauma, fractures, surgeries, and CTS, which together corresponded to more than 50% of the causes. 1 3 6 Most patients in the present study sought elective care (67%), scheduling surgical procedures on an outpatient basis, mainly for CTS (284 cases; 24%). The remainder (33%) were treated as urgent or emergent cases due to injuries requiring early intervention, such as fractures.

The different clinical conditions resulting in surgical intervention evaluated in the present study ( Table 3 ) fell into 3 broad groups: soft tissue trauma (43%), bone trauma (31.6%), and compressive syndromes (25.5%). In the statistical analysis of these general groups, only patients with compressive syndromes, mainly CTS, were twice as likely to develop CRPS during the postoperative period compared to any other condition. A review by A. Souza et al. 7 reported an incidence of 2% to 5% of CRPS after CTS decompression. These authors 7 also mentioned that chronic tunnel compression leads to hypersensitivity of the median nerve, local ischemia, and pain before treatment. For Gong et al., 8 intense pain before surgery is a significant risk factor for subsequent CRPS development, highlighting the importance of prior sensitization of the nervous system for pain. 8 Similarly, this prior stimulation in people with fibromyalgia scheduled for a surgical procedure for carpal tunnel decompression increased the CRPS risk by approximately twofold. 6 9

**Table 3:** General and specific diagnoses of the patients

*General diagnosis*	*Specific diagnosis*	*n*	*%*
*Bone trauma*	Hand bone fracture	110	9.3
	Forearm bone fracture	267	22.6
*Soft tissue trauma*	Synovial cyst	69	5.8
	De Quervain	29	2.5
	Trigger finger	98	8.3
	Hammer finger	27	2.3
	Dupuytren	16	1.4
	Material removal/debridement	42	3.6
	Adductor tenoplasty	15	1.3
	Tenorrhaphy	69	5.8
	Scaphoid pseudoarthrosis	15	1.3
	Miscellaneous	44	3.7
*Compressive syndrome*	Ulnar decompression	18	1.5
	Carpal tunnel syndrome	284	24
*Total*		1,183	100

Anesthetic preparation plays an essential role in the intraoperative period. In the present study, the predominant approach was intravenous general anesthesia (51.3%). As mentioned by A. Souza et al. 7 and Da Costa et al., 9 the anesthetic method, whether general intravenous or plexus block, does not increase the risk of developing postoperative CRPS. However, both studies 7 9 pointed to an association between the prolonged use of pneumatic tourniquets, regardless of the anesthetic technique, and the development of complex pain. Disproportionate pain in the postoperative period in the week after the traumatic event is another relevant predictive factor for the subsequent development of CRPS. 3 With this understanding, Da Costa et al. 9 consider plexus block a protective factor against CRPS, as it can reduce pain stimulation during surgery and maintain prolonged pain control in the postoperative period. 9

Another important consideration in the intraoperative setting involves incisions. Buller et al. 10 raised concerns about simultaneously performing surgeries for CTS and Dupuytren contracture. 10 They noted that this approach could slightly increase the likelihood of developing CRPS due to the length of the incisions required for both procedures. On the other hand, Rochlin et al. 11 reported a greater risk of CRPS in cases of extensive incisions or incisions performed on multiple fingers; however, they did not identify the specific relationship between the simultaneous performance of fasciotomy and carpal tunnel decompression described by Buller et al. 10 These authors reported that CRPS incidence does not change even when the procedures are performed in an open or endoscopic fashion. 10 11 In the present study, we observed that patients undergoing multiple incisions on the forearm and hand (7.6%) are 3 times more likely to develop CRPS, which is consistent with the literature findings on the increase in complex pain complications. 10 11

For Jo et al., 5 open fractures or those with significant instability tend to be more associated with the development of CRPS. They result from high-energy trauma with extensive damage to adjacent soft tissues and an intense concomitant inflammatory process. 5 DeGeorge et al. 12 observed that volar support plates used in unstable fractures or osteoporotic metaphyseal bones present complication rates ranging from 3% to 36%. The main complications included temporary paresthesias and acute CTS. These authors 12 did not record complex pain among the 647 surgeries evaluated. Most patients evaluated in the present research underwent decompression or tenorrhaphy procedures (63.7%). The treatment of most fractures was percutaneous (20.3%), that is, with low damage to soft tissues, which is consistent with the idea by Jo et al. 5 that the synthetic material is not directly responsible for CRPS progression, in contrast to previous trauma. 5

The incidence of CRPS after surgical procedures in our sample was low, which is in line with the literature. 1 3 5 12 The most relevant risk factors included previous diagnosis of compressive syndrome, especially CTS, and the performance of two or more incisions in the hands. We suggest a more detailed analysis of CTS decompression surgeries in future studies.

## Conclusion

When analyzing the surgical interventions, we concluded that performing two or more incisions substantially increased the risk of developing postoperative complex pain. The present study showed that CRPS is independent of the procedure, approach, synthetic material, or type of anesthesia. We strongly highlight the crucial role of sound preoperative planning and preventative measures to reduce the risk of developing CRPS.
