# Supplementary material for: Analysis of Surgical Procedures on the Forearm and Hand and Their Relationship with Complex Regional Pain Syndrome: A Cross-sectional Study
Source: Rev Bras Ortop (Sao Paulo). 2024 Jun 22;59(3):e403–8. doi: 10.1055/s-0044-1785659 (PMC11193584; doi:10.1055/s-0044-1785659)
Supplement: Related texts

# Research article: Análise dos procedimentos cirúrgicos no antebraço e na mão e sua relação com a síndrome da dor regional complexa: Um estudo transversal

**Keywords:** lesões da mão, procedimentos ortopédicos, síndrome do túnel do carpo, síndromes da dor regional complexa, síndromes de compressão nervosa

## Abstract

**Objetivo** A síndrome da dor regional complexa (SDRC) precisa ser mais bem compreendida. Assim, este estudo objetiva analisar se fatores pré e intraoperatórios poderiam estar relacionados ao desenvolvimento de SDRC no pós-operatório.

**Métodos** Foram revisados 1.183 prontuários de pacientes submetidos a cirurgias no antebraço e na mão entre 2015 e 2021. Os dados de interesse, como diagnóstico, incisões, material de síntese e anestesia realizada, foram coletados, tabulados e submetidos a testes estatísticos com posterior cálculo da razão de chances.

**Resultados** A maioria dos pacientes era do gênero feminino, com idade entre 30 e 59 anos, que buscaram o serviço de forma eletiva (67% dos casos). Os diagnósticos agrupados de forma geral foram: traumas de partes moles (43%), traumas ósseos (31,6%) e síndromes compressivas (25,5%). Durante esse período, 45 pacientes (3,8%) evoluíram com SDRC. A análise estatística mostrou que a chance de desenvolver SDRC é duas vezes maior em pacientes com síndrome compressiva, especialmente a síndrome do túnel do carpo (STC), que representou a maioria dos cirurgias realizadas em nosso serviço (24%). Em 7,6% dos casos, foram realizadas duas ou mais incisões, o que triplicou a possibilidade de SDRC pós-operatória. Gênero, idade, uso de material de síntese, ou tipo de anestesia não aumentaram estatisticamente o risco de SDRC no pós-operatório.

**Conclusão** Em suma, a incidência de SDRC é baixa, mas é importante conhecer e reconhecer os fatores de risco para a prevenção e a busca ativa no pós-operatório.

## Introdução

A síndrome da dor regional complexa (SDRC) é uma condição clínica caracterizada por dor desproporcional à injúria, persistente, associada a alterações de sensibilidade, de motricidade e de vascularização. Caso a condição perdure, leva a alterações de pele e atrofia no local acometido. 1 2 3 Ocorre predominantemente na porção periférica dos membros superiores, 2 mas pode acometer qualquer região do corpo. 1 2 3 4 Sua etiologia não é inteiramente compreendida; o diagnóstico é clínico, baseado nos critérios de Budapeste ( Tabela 1 ), e, até o momento, não há exames complementares específicos. 1 2 3 Ocorre geralmente após traumas, cirurgias ou condições que afetem o sistema nervoso local. 2 3 4 Acomete 2 a 4 vezes mais mulheres entre os 20 e 50 anos. 1 2 3 4 Os pacientes submetidos a cirurgia estão mais propensos a desenvolver SDRC do que aqueles tratados sem o procedimento. 3 5 Dessa forma, o objetivo deste estudo é analisar se as intervenções realizadas durante o ato cirúrgico, no tratamento das principais patologias da rotina do ambulatório de cirurgia da mão, contribuem ou não na evolução do paciente para o quadro de SDRC pós-operatória.

**Tabela 1:** Critérios clínicos de Budapeste para a síndrome de dor regional complexa

**Dor contínua desproporcional a qualquer evento desencadeante** **somada a pelo menos um sintoma, referido pelo paciente, em três das quatro categorias a seguir – sendo que um sintoma em pelo menos duas categorias devem sem evidenciados pelo profissional no momento da consulta**
** Sensorial: relatos de hiperalgesia e/ou alodinia**
** Vasomotor: relatos de diferença de temperatura e/ou alteração da cor da pele**
** Sudomotor/edema: relatos de edema e/ou alterações de sudorese e/ou** ** assimetria de sudorese**
** Motor/trófico: relatos de diminuição da amplitude de movimento e/ou** ** disfunção motora (fraqueza, tremor, distonia) e/ou alterações tróficas (cabelo,** ** unha, pele)**
**Não há outro diagnóstico que explique melhor os sinais e sintomas**

## Materiais e Métodos

Este estudo obteve aprovação dos Comitês de Ética das duas instituições em que os dados foram coletados, seguiu as diretrizes da instituição, e está em conformidade com a Resolução n ^o^ 466/2012 do Conselho Nacional de Saúde. Trata-se de um estudo transversal, observacional e retrospectivo, que incluiu todos os pacientes que passaram por procedimento cirúrgico entre janeiro de 2015 e dezembro de 2021. Essas cirurgias foram realizadas por um cirurgião de mão membro de uma equipe de ortopedia que atende uma regional de saúde. Após a alta hospitalar, os pacientes foram encaminhados ao ambulatório para acompanhamento com o mesmo profissional responsável pelo procedimento, a fim de garantir consistência na análise clínica. Este processo é especialmente relevante na SDRC devido aos seus diversos sinais e sintomas e à atual falta de um diagnóstico padrão-ouro, o que leva à necessidade de avaliações clínicas uniformes ao longo do acompanhamento médico. Como triagem para o diagnóstico, o profissional se guiou pelos critérios de Budapeste, segundo os quais o paciente deveria manifestar uma dor constante, não condizente com nenhum evento desencadeador, acompanhada de outros sinais e sintomas, conforme delineado na Tabela 1 .

Entre todos os pacientes operados, foram selecionados aqueles com diagnóstico de SDRC pelo menos trinta dias após a realização da cirurgia. Os pacientes com patologias tratadas de forma não cirúrgica, registros duplicados no sistema, ou pacientes com complicações, mas que não tinham sido submetidos a cirurgia durante o período pré-estabelecido para a pesquisa, foram excluídos.

Os dados foram coletados exclusivamente pela análise dos prontuários médicos. Foram registradas informações como gênero, idade, ano de atendimento, natureza do atendimento (eletivo ou de urgência), diagnóstico geral e específico, bem como informações específicas sobre o procedimento cirúrgico, como o número de incisões, os materiais de síntese usados, o tipo de anestesia administrada durante o procedimento e o subsequente desenvolvimento (ou não) da SDRC no pós-operatório.

As informações foram tabuladas no programa Excel (Microsoft Corp., Redmond, WA, Estados Unidos), versão 2013. Posteriormente, esses dados foram submetidos a análises estatísticas, com aplicação do teste do qui-quadrado seguida de análise de regressão logística univariada. As variáveis com resultados estatisticamente significativos foram incluídas na análise multivariada para identificar se havia associação independente delas com a SDRC. Os resultados foram expressos em razão de chances (RC) e em intervalos de confiança de 95% (IC95%), e a RC foi estabelecida quando o valor de *p* era inferior a 0,05 ( *p* < 0,05). Todas essas análises estatísticas foram conduzidas utilizando o aplicativo R (R Foundation for Statistical Computing, Viena, Áustria),versão 3.8.

Os dados quantitativos foram expressos em frequências e porcentagens para facilitar a compreensão. Em relação às informações qualitativas, os valores mais significativos são citados no texto e juntos aos dados gerais em uma tabela, para uma melhor análise e visualização dos dados durante a discussão.

## Resultados

No período em análise, 1.183 indivíduos foram operados, sendo 770 mulheres (65,1%) e 413 homens (34,9%), com idade média de 49,6 ± 17 anos. Conforme os critérios de Budapeste, 45 (3,8%) pacientes, com idade média de 53,02 ± 13 anos, desenvolveram SDRC durante o acompanhamento pós-operatório.

Com relação ao caráter do atendimento, a maioria dos pacientes buscou o serviço de forma eletiva, resultando em 795 procedimentos (67,2%), ao passo que as cirurgias de urgência, principalmente decorrente de fraturas, totalizaram 388 casos (32,8%).

Os pacientes submetidos a procedimentos cirúrgicos foram categorizados de acordo com a natureza da condição: lesões em partes moles (507; 42,9%), fraturas (374; 31,6%), e síndromes compressivas (302; 25,5%). Este último conjunto incluiu a síndrome do túnel do carpo (STC), que somou 284 procedimentos (24%), e a compressão do nervo ulnar, com 18 intervenções (1,5%). Depois dos procedimentos para STC, os mais frequentemente executados foram aqueles para fraturas dos ossos do antebraço, principalmente o rádio distal, com 267 cirurgias (22,6%), e para fraturas nos ossos da mão, com 110 casos (9,3%).

Ao contabilizar as incisões realizadas no mesmo paciente para a resolução do quadro patológico, a maioria somente necessitou de 1 acesso cirúrgico, num total de 1.093 pacientes (92,4%). Em contrapartida, em 90 casos (7,6%), foi necessário fazer 2 ou mais incisões.

Quanto aos materiais utilizados para a fixação, em 753 casos (63,7%) não se utilizou material de síntese rígida específico para realização do procedimento, incluindo-se aqui principalmente as descompressões e as tenorrafias. Os materiais mais frequentemente utilizados nas cirurgias foram os fios de Kirschner, em 240 (20,3%) casos, e as placas volares, em 126 (10,7%) casos.

Com relação à escolha da anestesia, predominou a aplicação de anestesia geral endovenosa, com um total de 607 procedimentos (51,3%) e, com um valor muito próximo, o bloqueio de plexo, em 576 pacientes (48,7%).

Com base na análise estatística, foram identificados fatores associados a um aumento do risco de desenvolver SDRC no pós-operatório. A presença de doença ou síndrome compressiva no paciente ( *p* = 0,0361), o diagnóstico específico de STC ( *p* = 0,0426) e a realização de 2 ou mais incisões cirúrgicas ( *p* = 0,0194) demonstraram estar relacionados ao aumento desse risco. Esses parâmetros foram então submetidos a uma análise logística multivariada, e o resultados foram que a síndrome compressiva (RC: 2; IC95%: 1,09–3,69), o diagnóstico de STC (RC: 1,98; IC95% 1,07–3,67) e a realização de 2 ou mais incisões (RC: 2,7; IC95% 1,26–6,18) apresentaram, de forma isolada, um aumento na chance de ocorrência de SDRC no pós-operatório. Um resumo dos resultados pode ser visto na Tabela 2 .

**Tabela 2:** Procedimentos cirúrgicos realizados e incidência de síndrome da dor regional complexa (SDCR)

		Pacientes: n(%)	Pacientes com SDRC: n(%)	Valor de *p* *(Qui-quadrado)*	*Razão de chances*	Intervalo de confiança de 95%
**Condição que levou ao procedimento cirúrgico**	Lesões em partes moles	507(42,9)	15(3)	0,074	−	−
	Fraturas	374(31,6)	12(3,2)			
	Síndromes compressivas	302(25,5)	18(6)	0,036	2	1,09–3,69
**Causas das cirurgias**	Síndrome do túnel do carpo	284(24)	17(6)	0,042	1,98	1,07–3,67
	Fratura dos ossos do antebraço	267(22,6)	11(4,1)	0,204	−	−
	Fratura dos ossos da mão	110(9,3)	1(0,9)			
	Dedo em gatilho	98(8,3)	4(4,1)			
	Retirada de material /desbridamento	80(6,8)	0			
**Número de incisões**	1	1093(92,4)	37(3,4)	0,019	2,7	1,26–6,18
	≥ 2	90(7,6)	8(8,9)			
**Material de síntese utilizado**	Nenhum	753(63,7)	32(4,2)	0,722	−	−
	Fio de Kirschner	240(20,3)	6(2,5)			
	Placa volar	126(10,7)	6(4,8)			
	Placa de compressão dinâmica	20(1,7)	1(5)			
	Parafuso Herbert	20(1,7)	0			
**Anestesia utilizada**	Geral endovenosa	607(51,3)	22(3,8)	1	1,01	0,56–1,83
	Bloqueio de plexo	576(48,7)	23(3,8)			

## Discussão

A SDRC é uma condição debilitante e complexa, que acomete principalmente a periferia dos membros superiores, tem predominância unilateral, e associa-se a alterações sensoriais, vasomotoras e distróficas. 1 2 4 Pode ser precedida por um lesão local conhecida, mas em 10% dos casos é idiopática. 1 3 5 6 Os sintomas geralmente iniciam de 15 a 30 dias após a injúria. 2 7 Seu diagnóstico é clínico e, até o momento, não há exames de comprovação validados. A literatura apresenta dados divergentes quanto à sua incidência, com estimativas abrangentes, 1 2 5 7 e quanto aos fatores de risco, que incluem comorbidades como depressão, fibromialgia, artrite reumatoide e distúrbios metabólicos. 1 2 3 4 5

Seu desenvolvimento ocorre em duas fases principais, cada uma com características próprias: na fase aguda ou quente, na qual ocorre a liberação de mediadores pró-inflamatórios, os sintomas incluem parestesias, hiperalgesia, anodinia, sinais de disfunção vasomotora, edema acentuado e discinesia; 1 3 6 e a fase crônica ou fria é caracterizada pela ativação de queratinócitos, fibroblastos e osteócitos, o que leva ao aumento da expressão de receptores adrenérgicos e à hiperestimulação do sistema nervoso simpático, o que ocasiona vasoconstrição local, com membros pálidos, frios e pegajosos. 1 3 6 Os sintomas muitas vezes são subjetivos e, se o processo não for interrompido, leva a sequelas como ausência de pelos, alterações nas unhas, atrofias musculares e dor crônica. 1 2 6

Dos pacientes operados durante os 5 anos de pesquisa (n = 1.183), 45 casos de SDRC (3,8%) foram diagnosticados no pós-operatório. Tanto o grupo total de pacientes operados quanto os que desenvolveram a síndrome eram compostos por uma maioria de mulheres com idade média entre 49 e 53 anos. 1 2 3 4 Embora Harden et al. 1 e Dutton e Rhee, 3 bem como outros autores, tenham destacado a prevalência de SDRC em mulheres (que chega a ser três vezes maior do que em homens), essa associação de gênero não mostrou aumentar o risco dessa complicação. 1 3 7

Estudos com múltiplos pacientes com SDRC, conforme mencionado por Taylor et al., 6 identificam a origem dessa dor crônica, e citam traumas, fraturas, cirurgias e STC, os quais, somados, correspondem a mais de 50% das causas. 1 3 6 Os pacientes deste estudo, em sua maioria, procuraram por atendimento de forma eletiva (67%), com procedimentos cirúrgicos agendados ambulatorialmente, sendo a causa mais prevalente a STC (284 casos; 24%). Os demais (33%) foram atendidos em regime de urgência ou emergência devido a lesões que exigiam intervenção precoce, como fraturas.

Os diferentes quadros clínicos que levaram à intervenção cirúrgica avaliados neste estudo ( Tabela 3 ) foram categorizados em 3 grupos amplos: traumas de partes moles (43%), traumas ósseos (31,6%) e síndromes compressivas (25,5%). Na análise estatística desses grupos gerais, apenas os pacientes que apresentaram síndromes compressivas, principalmente a STC, apresentavam uma probabilidade duas vezes maior de desenvolver a SDRC no pós-operatório em comparação com qualquer outra patologia operada. A revisão conduzida por A. Souza et al. 7 relata uma incidência de 2% a -5% de SDRC após a descompressão da STC. Esses autores 7 também mencionam que a compressão crônica do túnel leva a uma hipersensibilidade do nervo mediano, isquemia local e dor prévia ao tratamento. Conforme Gong et al., 8 a existência de dor intensa antes da cirurgia é um fator de risco significativo para o subsequente desenvolvimento da SDRC, destacando a importância da sensibilização prévia do sistema nervoso no surgimento do quadro de dor, 8 da mesma forma essa estimulação prévia, em pessoas com quadro de fibromialgia que vão passar pelo procedimento cirúrgico de descompressão do túnel do carpo, têm o potencial de aumentar o risco de SDRC em cerca de duas vezes. 6 9

**Tabela 3:** Diagnósticos gerais e específicos dos pacientes

*Diagnóstico geral*	*Diagnóstico específico*	*n*	*%*
*Trauma ósseo*	Fratura de ossos da mão	110	9.3
	Fratura de ossos do antebraço	267	22.6
*Trauma de partes moles*	Cisto sinovial	69	5.8
	De Quervain	29	2.5
	Dedo em gatilho	98	8.3
	Dedo em martelo	27	2.3
	Dupuytren	16	1.4
	Retirada de material/desbridamento	42	3.6
	Tenoplastia de adutor	15	1.3
	Tenorrafia	69	5.8
	Pseudoartrose de escafoide	15	1.3
	Outros	44	3.7
*Síndrome compressiva*	Descompressão ulnar	18	1.5
	Síndrome do túnel do carpo	284	24
*Total*		1.183	100

No período intra-operatório, a preparação anestésica desempenha um papel essencial. Neste estudo, a abordagem predominante foi a anestesia geral endovenosa (51,3%). Conforme mencionado por A. Souza et al. 7 e Da Costa et al., 9 independentemente do tipo de anestesia utilizado, seja geral endovenosa ou por bloqueio de plexo, não há aumento no risco de ocorrência de SDRC no pós-operatório. Entretanto, ambos os estudos 7 9 apontam uma associação entre o uso prolongado de garrotes pneumáticos, independentemente da técnica anestésica utilizada, e o desenvolvimento de dor complexa. Dor desproporcional no pós-operatório detectada na semana após o evento traumático também deve ser considerada fator preditivo relevante para o desenvolvimento posterior de SDRC. 3 Com esse entendimento, Da Costa et al. 9 consideram o bloqueio de plexo um fator protetor contra a SDRC, pois, é capaz de reduzir o estímulo de dor durante a cirurgia e manter um controle álgico prolongado no pós-operatório. 9

Outra consideração importante no cenário intraoperatório envolve as incisões. Buller et al. 10 mencionaram preocupação quanto à realização simultânea de cirurgias para STC e a contratura de Dupuytren; eles observaram que essa abordagem poderia aumentar discretamente a probabilidade de desenvolver SDRC devido à extensão das incisões necessárias para ambos os procedimentos. Por outro lado, Rochlin et al. 11 constataram que há um maior risco de desenvolver SDRC em casos de incisões extensas ou quando são realizadas incisões em múltiplos dedos da mão; entretanto, eles não identificaram a relação específica entre a realização simultânea de fasciotomia e de descompressão do túnel do carpo descrita por Buller et al., 10 e ainda complementaram acrescentando que a incidência não se altera nem mesmo quando os procedimentos são realizados por via aberta ou endoscópica. 10 11 Neste estudo, observamos que pacientes submetidos a múltiplas incisões no antebraço e na mão (7,6%) têm 3 vezes mais chances de desenvolver a SDRC, o que reforça os achados da literatura sobre o aumento das complicações de dor complexa nesse contexto. 10 11

Para Jo et al., 5 as fraturas expostas ou com instabilidade significativa tendem a estar mais associadas ao desenvolvimento de SDRC. Elas resultam de traumas de alta energia que causam danos extensos aos tecidos moles adjacentes e intenso processo inflamatório concomitante. 5 DeGeorge et al. 12 observaram que as placas de apoio volar, utilizadas em fraturas instáveis ou em ossos metafisários osteoporóticos, têm índices de complicação entre 3% e 36%, sendo as principais as parestesias temporárias e a STC aguda. Em seu estudo, 12 em que foram avaliadas 647 cirurgias, não foi registrado nenhum caso de dor complexa. A maioria dos pacientes avaliados no presente estudo foi submetida a procedimentos de descompressão ou tenorrafia (63,7%), e o tratamento da maior parte das fraturas foi percutâneo (20,3%), que são procedimentos com baixa agressão aos tecidos moles, o que se alinha à ideia de Jo et al., 5 de que o material de síntese não é diretamente responsável pela evolução para SDRC, mas o trauma pregresso.

A incidência de SDRC após procedimentos cirúrgicos em nossa amostra foi baixa, o que se alinha às descrições na literatura. 1 3 5 12 Os fatores de risco mais relevantes foram: diagnóstico prévio de síndrome compressiva, especialmente STC, e a realização de duas ou mais incisões nas mãos. Sugerimos análise mais detalhada de cirurgias de descompressão de STC em estudos futuros.

## Conclusão

Ao analisar as intervenções cirúrgicas neste estudo, concluímos que a realização de duas ou mais incisões aumentou substancialmente o risco de desenvolver dor complexa no pós-operatório, independentemente do procedimento, da via de acesso, ou do material de síntese utilizado, e não teve relação com a preferência anestésica para a cirurgia. Enfatizamos a importância crucial de um planejamento pré-operatório sólido e de medidas preventivas para reduzir o risco de desenvolver SDRC.
